# Horizontal transfer and the widespread presence of *Galileo* transposons in Drosophilidae (Insecta: Diptera)

**DOI:** 10.1590/1678-4685-GMB-2023-0143

**Published:** 2024-03-29

**Authors:** Henrique R.M. Antoniolli, Sebastián Pita, Maríndia Deprá, Vera L.S. Valente

**Affiliations:** 1 Universidade Federal do Rio Grande do Sul (UFRGS), Laboratório de Drosophila, Programa de Pós-Graduação em Genética e Biologia Molecular, Porto Alegre, RS, Brazil.; 2 Universidad de la República (UdelaR), Facultad de Ciencias, Sección Genética Evolutiva, Montevideo, Uruguay.

**Keywords:** DNA transposon, MITEs, *P* superfamily

## Abstract

*Galileo* is a transposon notoriously involved with inversions in *Drosophila buzzatii* by ectopic recombination. Although widespread in *Drosophila*, little is known about this transposon in other lineages of Drosophilidae. Here, the abundance of the canonical *Galileo* and its evolutionary history in Drosophilidae genomes was estimated and reconstructed across genera within its two subfamilies. Sequences of this transposon were masked in these genomes and their transposase sequences were recovered using BLASTn. Phylogenetic analyses were employed to reconstruct their evolutionary history and compare it to that of host genomes. *Galileo* was found in nearly all 163 species, however, only 37 harbored nearly complete transposase sequences. In the remaining, *Galileo* was found highly fragmented. Copies from related species were clustered, however horizontal transfer events were detected between the *melanogaster* and *montium* groups of *Drosophila*, and between the latter and the *Lordiphosa* genus. The similarity of sequences found in the *virilis* and *willistoni* groups of *Drosophila* was found to be a consequence of lineage sorting. Therefore, the evolution of *Galileo* is primarily marked by vertical transmission and long-term inactivation, mainly through the deletion of open reading frames. The latter has the potential to lead copies of this transposon to become miniature inverted-repeat transposable elements.

## Introduction

Transposable elements (TEs) belong to the repetitive fraction of genomes, and are linear sequences of DNA that have the ability to move within or between genomes (Wells and Feschotte, 2020). Classifications divide these sequences firstly into two classes, based on the intermediate molecule in their transposition process (Finnegan, 1989). Class I is composed of retrotransposons as their mobilization involves the synthesis of an RNA molecule, which are retrotranscribed into DNA and then inserted elsewhere in the genome (Wicker et al., 2007). On the other hand, the majority of Class II elements - or DNA transposons - are directly excised by their transposase (TPase), and then reinserted in another site in the genome (Wicker *et al*., 2007). 

In addition, TEs can be either autonomous or nonautonomous (Wicker et al., 2007). The first are those that present their structures preserved, encoding all necessary enzymes to be transposed. The latter comprise defective TEs that no longer encode nor produce their own proteins, and move only if recognized by the enzymes of a closely related autonomous TE; such as the Miniature Inverted-repeat Transposable Elements (MITEs). MITEs are non-autonomous TEs, derived from autonomous Class II transposons, and present a few structural characteristics: (i) small size, ranging from 50 to 500 base pairs (bp); (ii) AT-rich sequences; and (iii) a lack of a functional TPase (Deprá et al., 2012; Fattash et al., 2013).

Transposable elements are often referred to as “parasites” (Colonna Romano and Fanti, 2022), given their ability to invade new genomes and increase their copy number (Loreto et al., 2008). Horizontal transposon transfer (HTT) is the phenomenon in which a given TE “jumps” to the genome of a non-closely related species, i.e., sexually isolated organisms (Panaud, 2016). The role of HTT in shaping diversity as an endogenous source of evolution is widely recognized (Pace et al., 2008; Gilbert and Feschotte, 2018; Carvalho et al., 2023), and its frequency is much higher than previously thought (Schaack et al., 2010; Panaud, 2016; Peccoud et al., 2017; Melo and Wallau, 2020).

In this sense, several evolutionary events have been proposed as a direct consequence of TEs mobilization and/or recombination. For instance, in several taxa the variation and evolution of genome size are directly related to the amplification or contraction in TEs copy number (Canapa et al., 2015; Antoniolli et al., 2023). Nucleotide polymorphisms are also frequently produced after transposition events (Bourque et al., 2018). Transposable elements are also known to be related to changes in gene expression, either by silencing or enhancing them (Finnegan, 1989), and chromosomal rearrangements - i.e., deletions, duplications, translocations and inversions by ectopic recombination (Kidwell and Lisch, 1997). In the latter, distant loci in a genome carry highly similar TE copies, which allows homologous recombination to occur (see review in Bourque *et al*., 2018), thus resulting in a drastic modification in the chromosome architecture (Ren et al., 2018). Documented cases of a TE as a mediator of ectopic recombination include the families of retrotransposons *Bel-Pao*, *Doc*, *I* element and *roo*, as well as the transposons *foldback*, *Galileo* and *hobo* (Lim and Simmons, 1994; Delprat et al., 2009).

*Galileo* is a family of Class II transposons, and encodes its own TPase flanked by terminal inverted repeats (TIRs). Initially described as a *foldback*-like element, its TIRs and THAP domains exhibit similarities with those of the *P* element, leading to the classification of *Galileo* within the *P* superfamily (Marzo et al., 2008). However, unlike the *P* element, *Galileo* does not present introns (Marzo *et al*., 2008). *Galileo* was discovered by Cáceres et al. (1999) due to its association with the breakpoints of the *2j* inversion in wild specimens of *Drosophila buzzatii*. In fact, *Galileo* is the only TE known to induce chromosomal rearrangements in natural populations of *Drosophila* (Marzo *et al*., 2008), as most others have been observed in laboratory populations (Lim and Simmons, 1994). Besides the *2j* inversion, *Galileo* was involved with two other rearrangements described in *D. buzzatii* (Casals et al., 2003; Delprat et al., 2009). This makes this transposon as one of the most well-documented examples of a natural TE-induced chromosomal rearrangement. 

Studies have shown the widespread presence of *Galileo* in the *Drosophila* genus (Marzo et al., 2008; Acurio, 2015). The main focus of the present study was to characterize the evolutionary history of the *Galileo* family and evaluate its main transmission mode in Drosophilidae. This transposon was masked in genome assemblies of 163 species available at online databases, and TPase sequences found were employed for reconstructing a phylogeny and testing putative cases of HTT.

## Material and Methods

### Masking *Galileo* in the genome assemblies

Representative genome assemblies of 163 Drosophilidae species (see details on taxonomy and accession numbers in Table S1) were retrieved from GenBank (NCBI) with a Python package written by Blin (2021). These species belong to the *Chymomyza*, *Drosophila*, *Lordiphosa*, *Scaptodrosophila*, *Scaptomyza*, and *Zaprionus* genera of the Drosophilinae subfamily; and *Leucophenga* and *Phortica* of Steganinae subfamily (Table S1). BUSCO v.5 (Manni et al., 2021) was employed to assess the completeness of each assembly with the Diptera orthologous database.

The nucleotide sequence of seven *Galileo* copies characterized by Marzo et al. (2008) in *D. ananassae* (Dana\*Galileo -* BK006363), *D. buzzatii* (Dbuz\*Galileo* - EU334682 and EU334685), *D. mojavensis* (Dmoj\*Galileo -* BK006357), *D. persimilis* (Dper\*Galileo -* BK006361), *D. virilis* (Dvir\*Galileo* - BK006359) and *D. willistoni* (Dwil\*Galileo* - BK006360) were downloaded from GenBank, and used as queries in our workflow. Firstly, the queries were input as the repeat library in RepeatMasker (Smit *et al*., 2023) for masking canonical *Galileo* sequences in each genome assembly. The script ‘One code to find them all’ (Bailly-Bechet et al., 2014) was then employed to parse the output, recovering the nucleotide sequence of each identified copy in an assembly with at least 80% identity to its best query and a minimum length of 80 base pairs. 

### Phylogenetic analysis of *Galileo* potentially autonomous copies 

The complete nucleotide sequence encoding the transposase (TPase) of six copies (Dana\*Galileo*, Dbuz\*Galileo*, Dmoj\*Galileo*, Dper\*Galileo*, Dvir\*Galileo*, and Dwil\*Galileo*) served as queries for local BLASTn searches in each FASTA file containing the *Galileo* copies of each genome. Hits with at least 80% identity and coverage of at least 70% for any of the queries were used in downstream analyses. Additionally, a *P* element from the genome of *Drosophila buzzatii* (GenBank accession No. KC690135) and two copies of the *1360* element (GenBank accession Nos. AF533772 and AY138841) were included in the nucleotide matrix as outgroups. This matrix was aligned with MACSE v2 (Ranwez et al., 2018) in two steps: (i) using the option *alignSequences*, which aligns nucleotide sequences based on their underlying codon structure, accounting for frameshifts and stop codons; (ii) the resulting alignment was edited with the option *exportAlignment*, replacing codons containing frameshifts and internal stop codons with “N” (e.g., TG! was replaced by NNN). The codon alignment was then processed with Gblocks (Castresana, 2000) to remove poorly aligned regions, allowing the presence of gaps. 

The final codon alignment was translated to amino acids and used for a Bayesian phylogenetic inference (BI) analysis, performed in MrBayes 3.2.7 (Ronquist et al., 2012). The majority-rule consensus tree was built under the best amino acid substitution model, as estimated by ModelTest-NG (Darriba et al., 2020). Metropolis-coupled Markov chain Monte Carlo (MCMCMC) analysis was run with two parallel runs with four chains each for 1,000,000 generations, sampling every 100. Convergence was reached when the average standard deviation of split frequencies was below 1%. A burn-in of 25% was applied to the sampled trees before obtaining the consensus tree. The tree was visualized and edited in FigTree (Rambaut, 2018).

### Analysis of abundance and repeat profile

Forward short-reads of high-throughput whole genome sequencing were downloaded from the Sequence Read Archive of NCBI (see SRA accession No. in Table S1) for those species with positive hits for the TPase queries. These were submitted to the RepeatProfiler pipeline (Negm et al., 2021), an analysis in which sequencing reads are mapped against queries to build coverage graphs, allowing to infer which regions of a given query have a higher or lower abundance. 

Quality trim was performed with fastp (Chen et al., 2018), when reads had their adaptor removed while keeping only reads with no N base. The total reads were downsampled to 3 million, achieving near 1x coverage for all genomes (assuming a genome size mean of 200 megabases for species of the family Drosophilidae). In addition, five single-copy genes were randomly selected in the Diptera orthologous genes dataset of BUSCO 5 (Manni et al., 2021) to normalize the results (Table S2). The six complete copies of *Galileo* used in BLASTn searches were used as queries (Dbuz\*Galileo* - EU334685 was excluded because it was shorter than Dbuz\*Galileo* - EU334682). RepeatProfiler (Negm et al., 2021) was executed with default parameters.

### Inference of HTT events

Possible cases of HTT were determined based on incongruences between the phylogeny of host genomes and the phylogeny of *Galileo*. Validation of such cases was performed with the *vhica* R package (Wallau et al., 2015), implemented on the HTT-DB platform (Dotto et al., 2015). This method relies on discrepancies in the evolutionary rates of synonymous positions (dS), which considers codon usage bias (CUB), between nuclear genes (vertically transmitted) and transposable elements (TEs). Wallau *et al*. (2015) demonstrated that dS and CUB are correlated, and low values for both are indicative of inconsistencies with vertical transmission. 

Sequences of single-copy orthologous genes were searched in the assemblies with positive hits of *Galileo* using BUSCO 5 (Manni et al., 2021) and the Diptera orthologous database. Nucleotide sequences of 30 randomly selected genes (see Table S3) were aligned based on codons using the ClustalW algorithm (Thompson et al., 1994) implemented in MEGA 11 (Tamura et al., 2021). These alignments were used to compare the dS-CUB between the host nuclear genome and *Galileo* sequences. A substitution rate of 0.016 per million years (Sharp and Li, 1989) was applied to estimate the time of divergence between *Galileo* sequences.

To provide an evolutionary context for the results, a phylogenetic tree of the 37 host genomes was reconstructed using the entire set of BUSCO genes shared among them. Their amino acid sequences were aligned with MUSCLE (Edgar, 2004) and refined with trimAl (Capella-Gutiérrez et al., 2009), implemented in a pipeline written by McGowan (2020). *Scaptodrosophila lebanonensis* was included in this analysis as an outgroup. Their phylogenetic relationships were reconstructed under maximum likelihood in IQ-TREE 2 (Minh et al., 2020), with the best substitution model selected based on AIC scores (flags --*m* and --*merit*). Branch supports were estimated by applying 1,000 replicates of ultrafast bootstrap.

## Results

### Search for canonical *Galileo* copies 

Sequences of *Galileo* were masked in all analyzed genomes (Table S1), except for *D. ercepeae* and *D. nannoptera -* which belong to the *melanogaster* and *nannoptera* groups, respectively. Assemblies showed satisfactory levels of completeness, with the majority having more than 90% of single-copy orthologous genes (S). The exception was eight species, with S percentages ranging from 70% to 90% (see Table S4). In the second round of searches, conducted using local BLASTn with TPases as queries, 37 species yielded positive hits after the filtering process (Table S1). The positive results in the BLASTn search were limited to species within the *Drosophila* and *Lordiphosa* genera (Drosophilinae subfamily, Drosophilini tribe). All identified TPase sequences exhibited mutations, including stop codons, coding frame shifts, or both.

### Phylogenetic analysis and abundance of *Galileo* sequences 

The final sizes of nucleotide and amino acid alignments were 1,035 bp and 345 amino acids, respectively. The best amino acid substitution model was JTT+G4+F, based on the Akaike Information Criterion (AIC). Every copy of *Galileo* found in the genomes was placed in the same clade as its query. Major clades exhibited strong node support (PP > 0.95), with exceptions mainly observed among intraspecific sequences.

**Figure 1 -:**
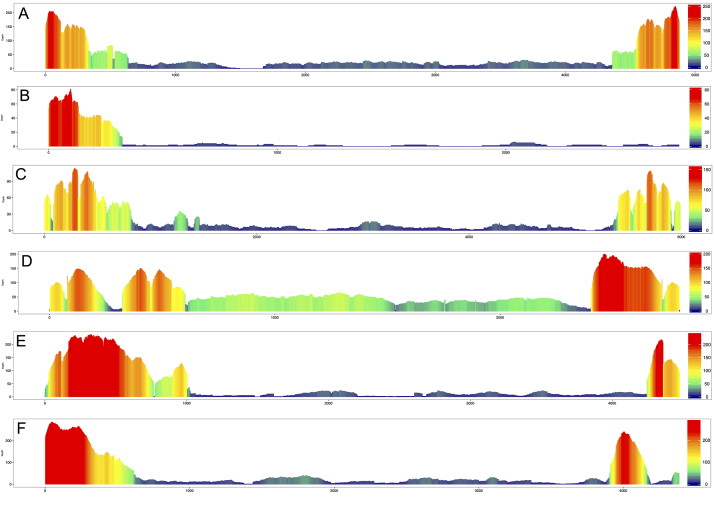
Coverage graphs for six queries of *Galileo* against its corresponding species: (A) Dana\*Galileo* in *Drosophila ananassae*; (B) Dbuz\*Galileo* in *D. buzzatii*; (C) Dmoj\*Galileo* in *D. mojavensis*; (D) Dper\*Galileo* in *D. persimilis*; (E) Dvir\*Galileo* in *D. virilis*; and (F) Dwil\*Galileo* in *D. willistoni*. Colors correspond to the coverage scale on the right side of each graph. Axis X corresponds to base pairs positions.

The query Dana\*Galileo* recovered three clades: the first two (yellow, Figure S1) containing sequences found in genomes of the *melanogaster* group (in which *D. ananassae* is phylogenetically placed); and the third (orange clade, Figure S1) containing sequences found in species of the *montium* group, along with *Lordiphosa collinella* and *L. stackelbergi* (pink sequences, Figure S1). Dwil\*Galileo* clustered homologous sequences found in the *willistoni* group (light pink sequences, Figure S1), along with its sister *saltans* group (blue sequences, Figure S1). On the other hand, the sequences of *Galileo* found by Dvir\*Galileo* (green clade, Figure S1) in species of the *virilis* group formed a sister clade (PP = 1.0) to those of the *willistoni* and *saltans* groups. Finally, Dper\*Galileo* recovered *Galileo* from species belonging to the *obscura* group (red clade, Figure S1), and Dmoj\*Galileo* retrieved sequences in *D. mojavensis* (purple clade, Figure S1). The abundance of *Galileo* sequences in these species, as assessed by the coverage analysis in RepeatProfiler, showed that the TPase region had lower coverage than that of TIRs in all cases (Figures 1 and S2-S7).

### Inference of HTT events

**Figure 2 -:**
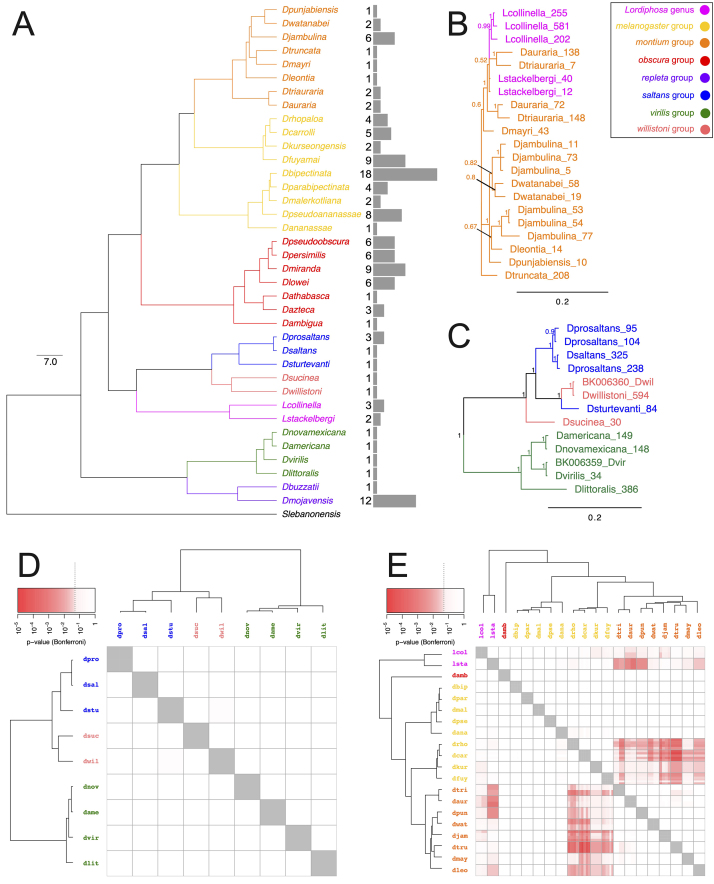
(A) Ultrametric tree showing the phylogenetic relationships between species harboring nearly complete transposases, assessed through maximum likelihood. Ultrafast bootstrap (UFboot) not shown, as for all nodes UFboot = 100. (B and C) Majority-rule consensus tree showing the phylogenetic relationships between sequences of *Galileo*, (B) found in genomes of the *montium* group of *Drosophila* and species of the *Lordiphosa* genus, and (C) found in genomes of the *saltans*, *virilis* and *willistoni* groups of *Drosophila*; numbers next to each node reflect its posterior probability support. (D and E) Results of the horizontal transposon transfer (HTT) analysis in vhica, between (D) *saltans*, *virilis* and *willistoni* groups of *Drosophila*; and (E) *Lordiphosa* genus and *melanogaster* and *montium* groups of *Drosophila*. (D and E) Red squares represent statistically significant (P < 0.05) pairwise comparisons between sequences of *Galileo*, indicating a HTT event. Phylogenetic relationships between host genomes are shown by ultrametric trees drawn on the external sides of each graph.

Two major incongruences were found between host species (Figure 2A) and *Galileo* phylogenies. The first is the similarity of elements found in *Lordiphosa collinella* and *Lordiphosa stackelbergi* with species of the *montium* group (Figure 2B). The second incongruence (Figure 2C) is the clade formed by *virilis* (*Drosophila* subgenus) and *willistoni* plus *saltans* groups (*Sophophora* subgenus). No signals of HTT events were detected (p-value > 0.05) between the species of the *virilis* group and the *willistoni* and *saltans* groups (Figure 2D). However, HTT was detected (p-value < 0.05) between *L. collinella* and *L. stackelbergi* and species of the *montium* group. Signals were also detected between the *melanogaster* and *montium* groups, both belonging to the *Sophophora* subgenus (Figure 2E). Estimates of divergence times (Table S5) span from ~679 thousand years ago (*D. auraria* x *L. stackelbergi*) to ~6 million years ago (*D. carrolli* × *D. watanabei*).

## Discussion

The 163 genomes analyzed in this study provided a broader sampling across Drosophilidae when compared to previous studies (Marzo et al., 2008; Acurio, 2015), including many different taxonomic levels. We were able to search for *Galileo* in the genomes of the two subfamilies - Drosophilinae and, for the first time, Steganinae. Furthermore, our sampling included two tribes of the first (Colocasiomyini and Drosophilini) and two tribes of the latter (Gitonini and Steganini). Indeed, the *Drosophila* genus is a paraphyletic lineage due to the offshoot of several genera within its phylogenetic tree (Suvorov et al., 2022); e.g., the *Lordiphosa* genus is placed within the *Sophophora* subgenus as a sister lineage to the Neotropical clade, which includes the *saltans* and *willistoni* groups (Figure 2D).

### *Galileo* is fragmentally widespread in Drosophilidae 

The majority of *Galileo* sequences recovered in our study consisted of fragments. Indeed, high levels of structural dynamism in *Galileo* have been described both within and between genomes, as TIRs presented variable sizes (see review in Marzo et al., 2008). Therefore, our results suggest that the canonical *Galileo* is widespread and abundant in the genomes of Drosophilidae, although its copies are potentially defective. Given the lack of coding for a transposase, these copies would be incapable of autonomous transposition, remaining as relics - as in the case of Miniature Inverted-repeat Transposable Elements (MITEs). 

The hypothesis of classifying these fragmented copies as MITEs of *Galileo* in *D. mojavensis* was considered by Marzo et al. (2013a), but was discarded by those authors because the sequences were longer and had a lower copy number compared to typical MITEs. However, our analysis of normalized coverage suggested the opposite; highly amplified short segments of *Galileo* TIRs were detected (Figures 1 and S2-S7), consistent with the size of MITEs. In *D. virilis*, for example, the TPase segment of Dvir\*Galileo* had a low coverage (~10X) while its TIRs had a coverage of < 200X (Figure 1E). Although strong evidence was found, further characterization is still needed to assist in the classification of these short canonical sequences as MITEs.

**Figure 3 -:**
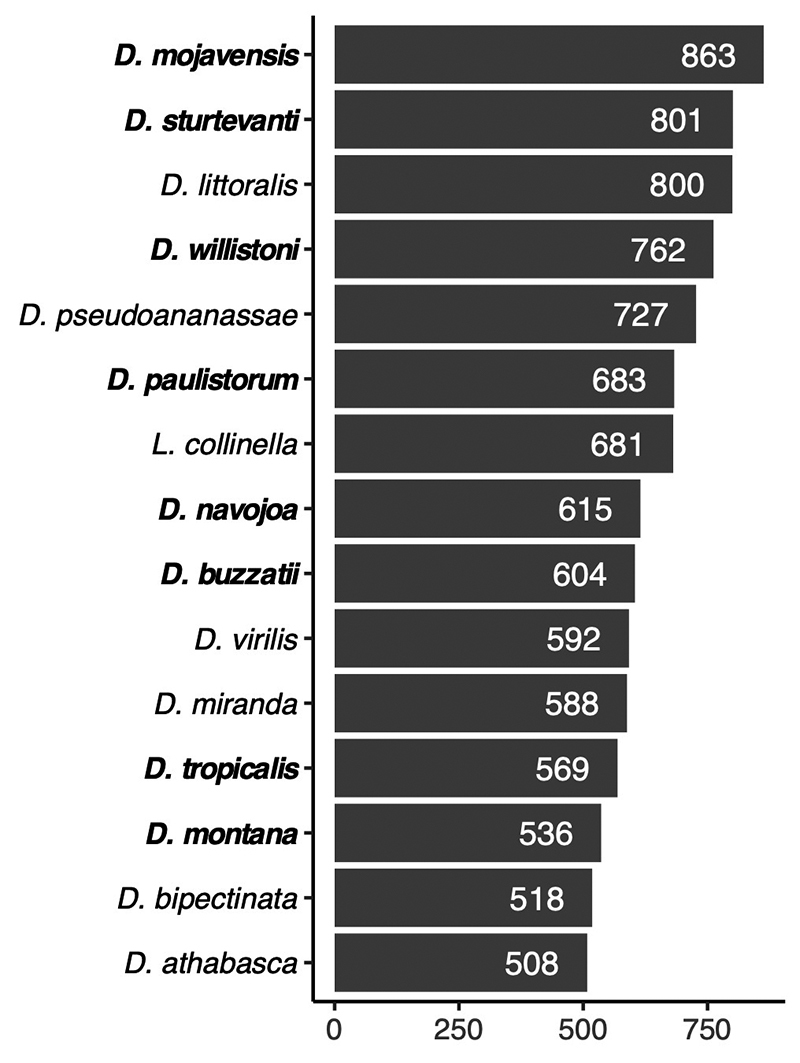
Figure 3 - Number of sequences (X axis) masked as *Galileo* elements by RepeatMasker for the top 15 species (Y axis) with the highest number of sequences. Species highlighted in bold are endemic to the Neotropical region.

Interestingly, *Galileo* seems to be highly amplified in Neotropical species. Among the 15 species with the highest copy number (Figure 3; Table S1), eight are endemic to the Neotropical region: *D. mojavensis*, *D. sturtevanti*, *D. willistoni*, *D. paulistorum*, *D. navojoa*, and *D. buzzatii*, *D. tropicalis*, and *D. montana* (listed from the highest to the lowest copy number). In fact, the heterogeneity found across the Neotropical region provides innumerous distinct environments, challenging the survival of species (Miranda et al., 2022). Such environments also impact genomes, as expanding into new areas may relieve the epigenetic silencing or control of TEs, leading to their mobilization and amplification (Gregory, 2001; Rebollo et al., 2010; Antoniolli et al., 2023). For instance, *D. willistoni* - which harbors an exceptional diversity of *Galileo* (Gonçalves et al., 2014) - is distributed throughout the Neotropical region, and TEs differentially populate its genomes (Bertocchi et al., 2022). 

### Signals of HTT in the *Sophophora* subgenus 

The overall congruence between the phylogeny of *Galileo* and that of its host genomes, in terms of clustering species of the same group into the same clade (Figure S1), may be explained by vertical transmission (Acurio, 2015). However, the observed incongruence involving copies found in *Lordiphosa* and species of the *montium* group (Figure 2B) was confirmed as horizontal transfer (HTT) event (Figure 2E). The *Lordiphosa* genus is actually a sister lineage to the *willistoni* group, and its MRCA with the *montium* group diverged around 40 million years ago (Mya) (Suvorov et al., 2022). In this case, the oldest HTT event between them (*L. stacklbergi* × *D. punjabiensis*) is estimated to have occurred at around 2.2 Mya; much more recent than their MRCA.

Other cases of HTT involved the *melanogaster* and *montium* groups, whose MRCA diverged around 20 Mya (Suvorov et al., 2022); also much older than the oldest HTT detected between them (around 6 Mya for *D. carrolli* × *D. watanabei*). The species involved with HTT events occur in sympatry, mainly in the Palearctic region of Asia (TaxoDros v1.04) - which permits a niche overlap. Addittionaly, *Galileo* exhibits a patchy distribution both in the *Lordiphosa* genus and the *melanogaster* and *montium* groups (Table S1); in this case, the TE is present in some species but absent in another closely related one(s). 

Furthermore, a specific THAP binding site for the *Galileo* transposase was identified at the 3’ end TIRs (Marzo et al., 2013b). The sequences of *Galileo* found in these species involved in HTT cases presented highly conserved and amplified 3’ TIRs (Figures 1 and S2-S7), providing further support for the plausibility of such HTT events. Nonetheless, the successful establishment of a TE in new genomes is highly dependent on its transposition rate (Le Rouzic and Capy, 2005), as it must avoid being lost in the population due to genetic drift (Blumenstiel, 2019). While *L. stackelbergi* presented a low number of sequences (49 sequences), *L. collinella* harbors more than 680 sequences (Table S1), similar to *D. buzzatii* (604 sequences), in which *Galileo* was first described. Many other cases of low copy number were also detected (Table S1), and the smallest include *D. ambigua* (10), *D. punjabiensis* (37). and *D. watanabei* (58). The process of stochastic loss of an element may explain both its patchy distribution and low copy number (Blumenstiel, 2019), as observed in *mariner*-like elements in *Drosophila* (Lohe et al., 1995) and *Rex* elements in the ray-finned fish *Characidium* (Pucci et al., 2018).

### Lineage sorting explains the similarity between the *saltans*, *virilis* and *willistoni* groups 

Marzo et al. (2008) described a high similarity between the copies found in the genomes of *D. virilis* and *D. willistoni*. Interestingly, the first belongs to the *Drosophila* subgenus, while the latter belongs to the *Sophophora* subgenus - their MRCA diverged around 49.9 Mya (Suvorov et al., 2022). Acurio (2015) later confirmed this close relationship, identifying it along with the *guarani* and *tripunctata* groups (*Drosophila* subgenus). Our results further corroborate both studies by expanding the sample size to include *D. littoralis* and *D. novamexicana* (*virilis* group).

Interestingly, *Galileo* sequences found in each of these two groups clustered into sister clades that corresponded to their host species, with the addition of sequences from the *saltans* group in the latter. This clade (*virilis* + *saltans* + *willistoni*) was the first to split in the evolution of *Galileo* - also congruent with Marzo et al. (2008). These authors also proposed two explanations for the incongruence between the phylogenies of *Galileo* and its host genomes: lineage sorting with ancestral HTT (Acurio, 2015); or horizontal transfer itself. As no signal of HTT was detected between or within these three species groups (Figure 2A), lineage sorting is a plausible explanation (Cummings, 1994). In this case, the transposon is vertically transmitted, but its copies coalesce prior to the split between the host species (Tenaillon et al., 2010) or are differentially lost along the branches of the species tree (Marzo *et al*., 2008).

## Conclusions

The evolutionary history of *Galileo* in Drosophilidae is marked mostly by vertical and possibly ancient horizontal transmissions, as identified by Acurio (2015), with stochastic loss through genetic drift occurring while species diverged. In addition, its high fragmentation level is compatible with the characteristics of MITEs, although a thorough characterization is still needed to confirm this. *Galileo* found favorable conditions for its amplification in the heterogeneous Neotropical region, with an astounding copy number detected in Drosophilidae species inhabiting this area. Finally, considering the potential of *Galileo* to induce chromosomal rearrangements and their evolutionary implications, the HTT described between *Lordiphosa* and the *montium* group, and between the latter and the *melanogaster* group, these results raise an intriguing question (Alfredo Ruiz, personal communication): could evolution be infectious? 

## Supplementary material

The following online material is available for this article:

Figure S1 - Phylogenetic relationships between sequences of *Galileo* found across genomes of Drosophilidae, reconstructed through Bayesian Inference.

Figure S2 - Normalized coverage graphs for Dana\*Galileo* used for searching transposase sequences across genomes of Drosophilidae.

Figure S3 - Normalized coverage graphs for Dbuz\*Galileo* used for searching transposase sequences across genomes of Drosophilidae.

Figure S4 - Normalized coverage graphs for Dmoj\*Galileo* used for searching transposase sequences across genomes of Drosophilidae.

Figure S5 - Normalized coverage graphs for Dper\*Galileo* used for searching transposase sequences across genomes of Drosophilidae.

Figure S6 - Normalized coverage graphs for Dvir\*Galileo* used for searching transposase sequences across genomes of Drosophilidae.

Figure S7 - Normalized coverage graphs for Dwil\*Galileo* used for searching transposase sequences across genomes of Drosophilidae.

Table S1 - List and taxonomy of Drosophilidae species included in this study, including positive results from BLASTn searches of the *Galileo* transposase sequences and accession numbers to the genome assembly and short-read sequencing data on NCBI.

Table S2 - List of genes used to normalize the results of profile and abundance of *Galileo* across the analyzed genomes in this study.

Table S3 - List of genes used for Codon Usage Bias (CUB) comparisons in the analysis of horizontal transposon transfer (HTT) in vhica R package.

Table S4 - Statistics of assembly completeness for each analyzed genome.

Table S5 - Statistically significant results of pairwise comparisons of horizontal transposon transfer performed with vhica at HTT-DB.
