# Supplementary figures and images for: Horizontal transfer and the widespread presence of Galileo transposons in Drosophilidae (Insecta: Diptera)
Source: Genet Mol Biol. 2024 Mar 29;46(3 Suppl 1):e20230143. doi: 10.1590/1678-4685-GMB-2023-0143 (PMC10990002; doi:10.1590/1678-4685-GMB-2023-0143)

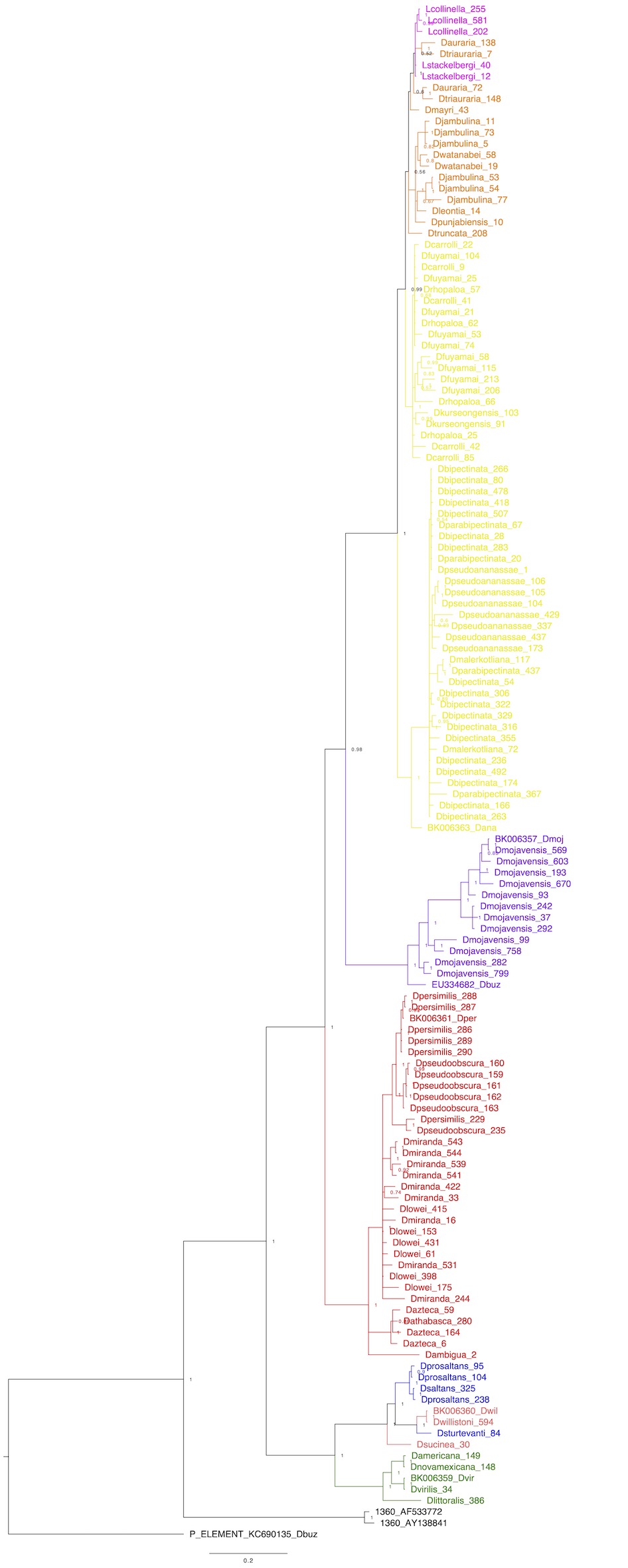

Supplement: Figure S1 - [file 1415-4757-GMB-46-3-s1-e20230143-s1.zip › Suppl_FigureS1.jpg]

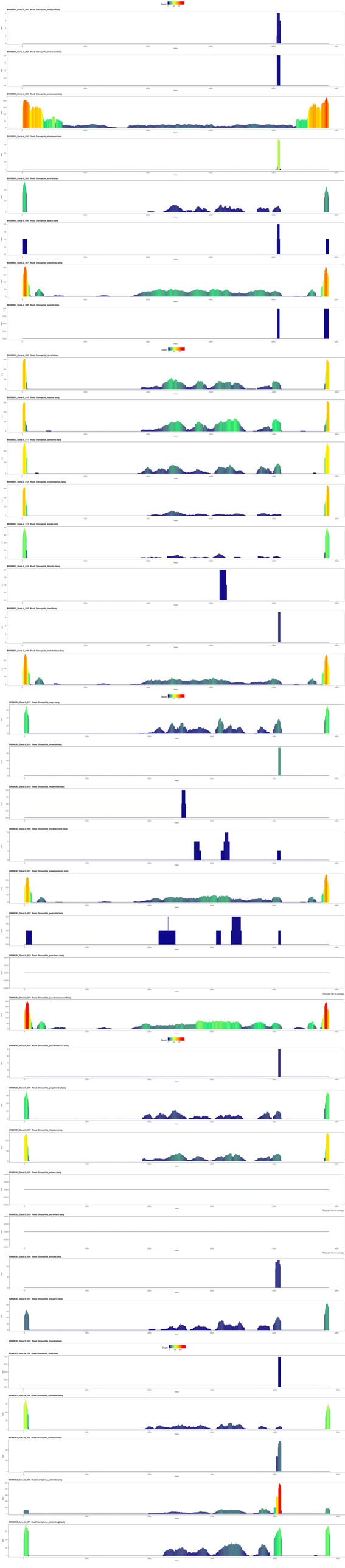

Supplement: Figure S2 - [file 1415-4757-GMB-46-3-s1-e20230143-s2.zip › Suppl_FigureS2.jpeg]

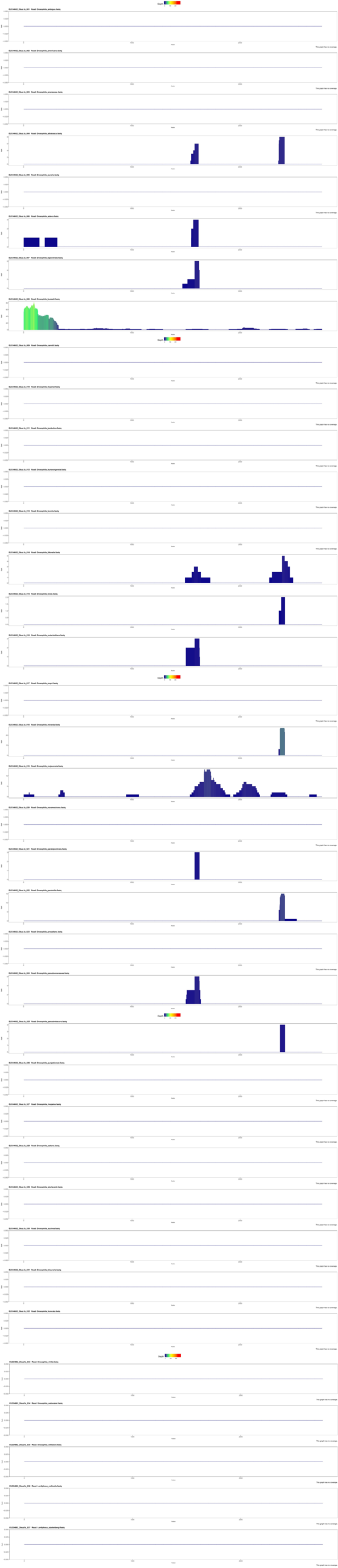

Supplement: Figure S3 - [file 1415-4757-GMB-46-3-s1-e20230143-s3.zip › Suppl_FigureS3.jpeg]

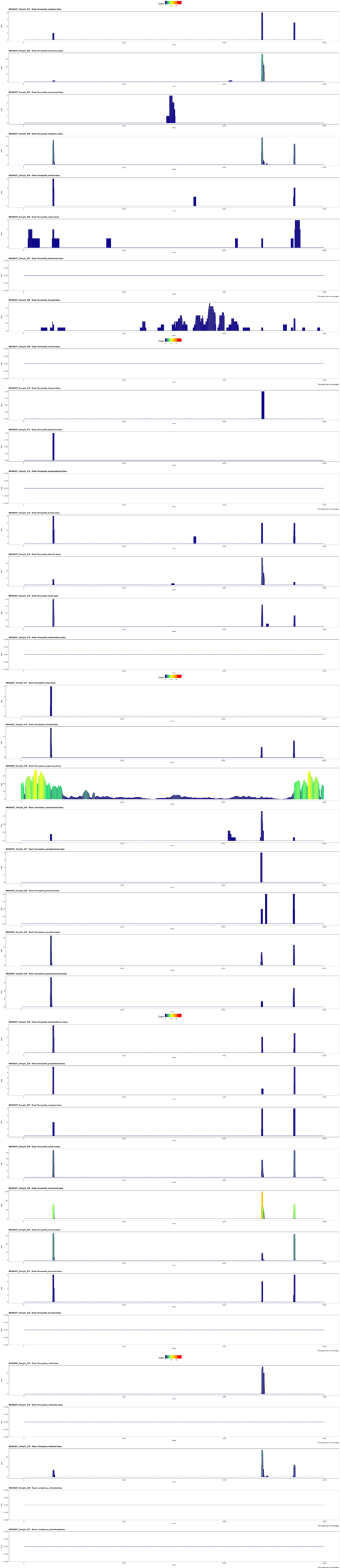

Supplement: Figure S4 - [file 1415-4757-GMB-46-3-s1-e20230143-s4.zip › Suppl_FigureS4.jpeg]

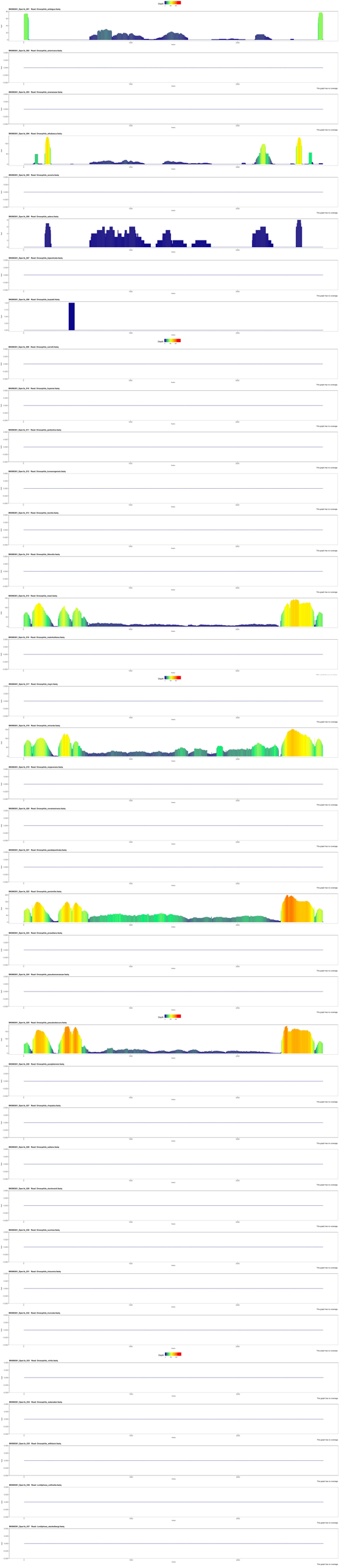

Supplement: Figure S5 - [file 1415-4757-GMB-46-3-s1-e20230143-s5.zip › Suppl_FigureS5.jpeg]

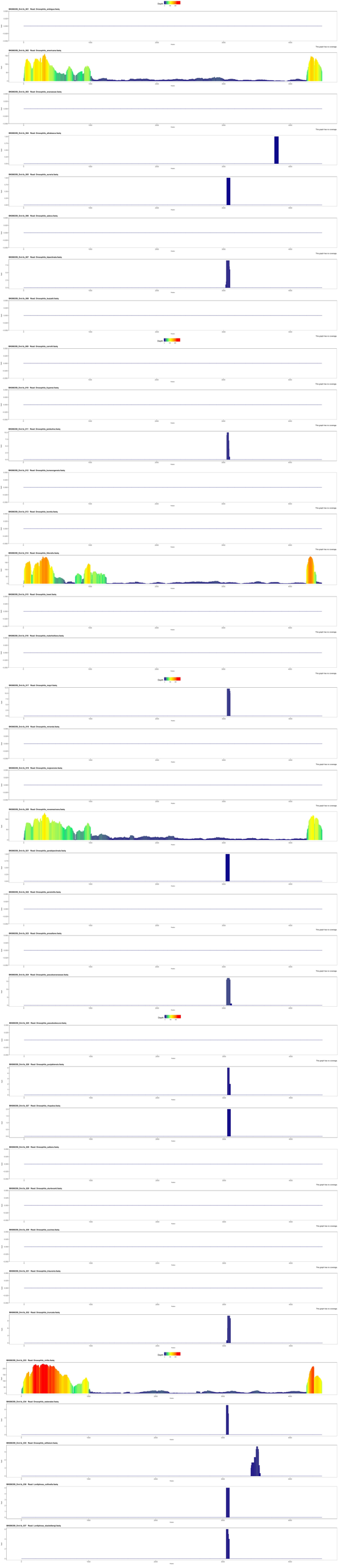

Supplement: Figure S6 - [file 1415-4757-GMB-46-3-s1-e20230143-s6.zip › Suppl_FigureS6.jpeg]

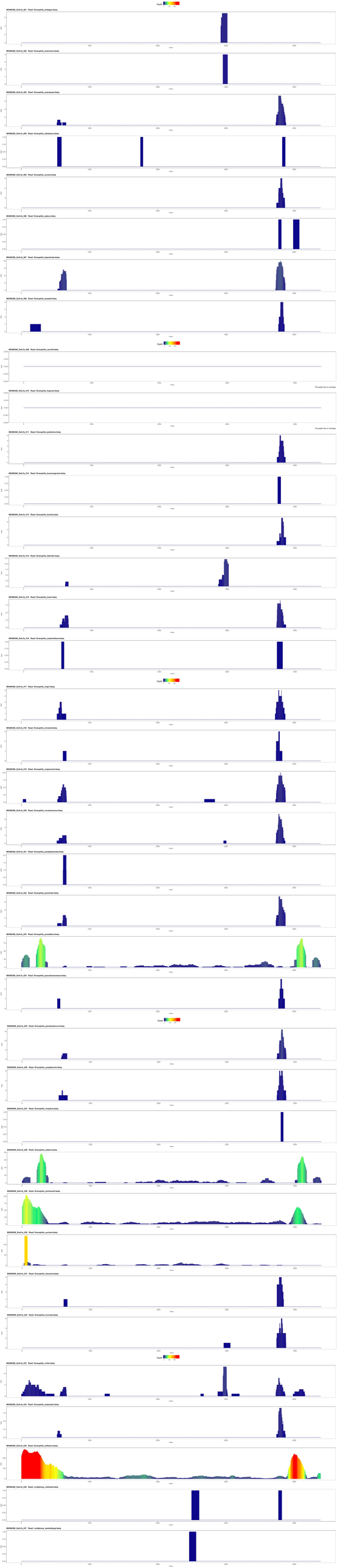

Supplement: Figure S7 - [file 1415-4757-GMB-46-3-s1-e20230143-s7.zip › Suppl_FigureS7.jpeg]
