# Supplementary material for: Horizontal transfer and the widespread presence of Galileo transposons in Drosophilidae (Insecta: Diptera)
Source: Genet Mol Biol. 2024 Mar 29;46(3 Suppl 1):e20230143. doi: 10.1590/1678-4685-GMB-2023-0143 (PMC10990002; doi:10.1590/1678-4685-GMB-2023-0143)
Supplement: Figure S6 - [file 1415-4757-GMB-46-3-s1-e20230143-s6.zip › gmb-2023-0143_20240228_suppl6.pdf]

Genetics and Molecular Biology
https://doi.org/10.1590/1678-4685-GMB-2023-0143

Supplementary Material to "Horizontal transfer and the widespread presence of *Galileo* transposons in Drosophilidae (Insecta: Diptera)"

Figure S6 – Normalized coverage graphs for Dvir\Galileo used for searching transposase sequences across genomes of Drosophilidae. Species are identified on the top left of each graph.
