# Supplementary material for: Horizontal transfer and the widespread presence of Galileo transposons in Drosophilidae (Insecta: Diptera)
Source: Genet Mol Biol. 2024 Mar 29;46(3 Suppl 1):e20230143. doi: 10.1590/1678-4685-GMB-2023-0143 (PMC10990002; doi:10.1590/1678-4685-GMB-2023-0143)
Supplement: Table S1 - [file 1415-4757-GMB-46-3-s1-e20230143-s8.pdf]

Genetics and Molecular Biology
https://doi.org/10.1590/1678-4685-GMB-2023-0143

Supplementary Material to "Horizontal transfer and the widespread presence of *Galileo* transposons in Drosophilidae (Insecta: Diptera)"

Table S1 – List and taxonomy of Drosophilidae species included in this study, including positive results from BLASTn searches of the *Galileo* transposase sequences and accession numbers to the genome assembly and short-read sequencing data on NCBI.

Subfamily	Tribe	Genus	Subgenus or lineage	Species group	Species	RepeatMasker	TPase	N of seqs	GenBank
Drosophilinae	Colocasiomyini	*Chymomyza*	--	*costata*	*Chymomyza costata*	yes	no	25	GCA_018150985
		Scaptodrosophila	--	*victoria*	*Scaptodrosophila lebanonensis*	yes	no	386	GCA_003285725
	Drosophilini	*Drosophila*	*Dorsilopha*	*busckii*	*Drosophila busckii*	yes	no	37	GCA_011750605
			Drosophila	--	*Drosophila pruinosa*	yes	no	105	GCA_018150935
				cardini	*Drosophila arawakana*	yes	no	44	GCA_018151165
					Drosophila cardini	yes	no	156	GCA_018903735
					Drosophila dunni	yes	no	104	GCA_018152125
					Drosophila nigrodunni	yes	no	28	GCA_020829145
				funebris	*Drosophila funebris*	yes	no	39	GCA_018901825
				immigrans	*Drosophila albomicans*	yes	no	48	GCA_009650485
					Drosophila immigrans	yes	no	33	GCA_018153375
					Drosophila kohkoa	yes	no	35	GCA_019972355
					Drosophila nasuta	yes	no	56	GCA_019972435
					Drosophila neonasuta	yes	no	9	GCA_005889595

					Drosophila quadrilineata	yes	no	218	GCA_018150725
					Drosophila rubida	yes	no	20	GCA_021223945
					Drosophila sulfurigaster	yes	no	31	GCA_021223965
				melanica	*Drosophila melanica*	yes	no	17	GCA_004143765
					Drosophila micromelanica	yes	no	25	GCA_004143825
					Drosophila nigromelanica	yes	no	26	GCA_004149445
				nannoptera	*Drosophila nannoptera*	no	no	0	GCA_020883555
					Drosophila pachea	yes	no	1	GCA_020883565
				quinaria	*Drosophila innubila*	yes	no	104	GCA_004354385
				repleta	*Drosophila arizonae*	yes	no	437	GCA_001654025
					Drosophila buzzatii	yes	yes	604	*SRR12849626
Drosophilinae	Drosophilini	*Drosophila*	*Drosophila*	*repleta*	*Drosophila hydei*	yes	no	28	GCA_003285905
					Drosophila mojavensis	yes	yes	863	GCA_018153725; SRR12849565
					Drosophila navojoa	yes	no	615	GCA_001654015
					Drosophila repleta	yes	no	70	GCA_018903745
				robusta	*Drosophila lacertosa*	yes	no	20	GCA_004143845
					Drosophila robusta	yes	no	19	GCA_004143805
				tumiditarsus	*Drosophila repletoides*	yes	no	24	GCA_018150835
				virilis	*Drosophila americana*	yes	yes	462	GCA_018152915; SRR13070711
					Drosophila littoralis	yes	yes	800	GCA_018903485; SRR13070670
					Drosophila montana	yes	no	536	GCA_003086615
					Drosophila novamexicana	yes	yes	453	GCA_003285875; SRR20226096

					Drosophila virilis	yes	yes	592	GCA_003285735; SRR6426000
			Hawaiian	*grimshawi*	*Drosophila grimshawi*	yes	no	26	GCA_018153295
					Drosophila murphyi	yes	no	24	GCA_018904325
					Drosophila sproati	yes	no	20	GCA_018904355
			Sophophora	*melanogaster*	*Drosophila ananassae*	yes	yes	383	GCA_017639315; SRR6425991
					Drosophila biarmipes	yes	no	16	GCA_018148935
					Drosophila bipectinata	yes	yes	518	GCA_018153845; SRR6425989
					Drosophila carrolli	yes	yes	168	GCA_018152295; SRR13070705
					Drosophila elegans	yes	no	27	GCA_018152505
					Drosophila ercepeae	no	no	0	GCA_018150545
					Drosophila erecta	yes	no	107	GCA_003286155
					Drosophila eugracilis	yes	no	12	GCA_018153835
					Drosophila ficusphila	yes	no	3	GCA_018152265
					Drosophila fuyamai	yes	yes	284	GCA_018153365; SRR13070718
					Drosophila gunungcola	yes	no	33	GCA_011057485
Drosophilinae	Drosophilini	*Drosophila*	*Sophophora*	*melanogaster*	*Drosophila ironensis*	yes	no	14	GCA_021223825
					Drosophila kurseongensis	yes	yes	104	GCA_018153305; SRR13070719
					Drosophila malerkotliana	yes	yes	372	GCA_018153235; SRR13070717
					Drosophila mauritiana	yes	no	65	GCA_004382145
					Drosophila melanogaster	yes	no	61	GCA_000001215
					Drosophila orena	yes	no	190	GCA_005876975
					Drosophila oshimai	yes	no	3	GCA_018150695

					Drosophila pandora	yes	no	422	GCA_021223865
					Drosophila parabipectinata	yes	yes	460	GCA_018153455; SRR13070721
					Drosophila pseudoananassae	yes	yes	727	GCA_018153035; SRR13070714
					Drosophila pseudotakahashii	yes	no	22	GCA_021223935
					Drosophila rhopaloa	yes	yes	146	GCA_018152115; SRR345538
					Drosophila santomea	yes	no	53	GCA_016746245
					Drosophila sechellia	yes	no	76	GCA_004382195
					Drosophila simulans	yes	no	23	GCA_016746395
					Drosophila subpulchrella	yes	no	31	GCA_014743375
					Drosophila suzukii	yes	no	35	GCA_013340165
					Drosophila takahashii	yes	no	8	GCA_018152695
					Drosophila teissieri	yes	no	65	GCA_016746235
					Drosophila varians	yes	no	38	GCA_018150405
					Drosophila yakuba	yes	no	52	GCA_016746365
				montium	*Drosophila anomelani*	yes	no	29	GCA_018905855
					Drosophila asahinai	yes	no	69	GCA_008042795
					Drosophila auraria	yes	yes	198	GCA_008042615; SRR9997924
					Drosophila baimaii	yes	no	40	GCA_018874675
					Drosophila bakoue	yes	no	26	GCA_008044335
					Drosophila barbarae	yes	no	14	GCA_018874595
Drosophilinae	Drosophilini	*Drosophila*	*Sophophora*	*montium*	*Drosophila birchii*	yes	no	27	GCA_008042755
					Drosophila bocki	yes	no	26	GCA_008042715
					Drosophila bocqueti	yes	no	26	GCA_018151655

					Drosophila bunnanda	yes	no	3	GCA_021223745
					Drosophila burlai	yes	no	33	GCA_008042655
					Drosophila chauvacae	yes	no	12	GCA_018905695
					Drosophila diplacantha	yes	no	9	GCA_018874655
					Drosophila fengkainensis	yes	no	24	GCA_018874535
					Drosophila greeni	yes	no	33	GCA_018874515
					Drosophila jambulina	yes	yes	83	GCA_018152175; SRR13070704
					Drosophila kanapiae	yes	no	71	GCA_008042475
					Drosophila kikkawai	yes	no	32	GCA_018152535
					Drosophila lacteicornis	yes	no	73	GCA_008044355
					Drosophila leontia	yes	yes	25	GCA_008042735; SRR9997936
					Drosophila lini	yes	no	28	GCA_018905715
					Drosophila malagassya	yes	no	6	GCA_018874475
					Drosophila mayri	yes	yes	51	GCA_008042485; SRR9997930
					Drosophila nikananu	yes	no	301	GCA_008042635
					Drosophila ogumai	yes	no	32	GCA_018904815
					Drosophila ohnishii	yes	no	30	GCA_018905755
					Drosophila orosa	yes	no	25	GCA_018904745
					Drosophila parvula	yes	no	111	GCA_018904695
					Drosophila pectinifera	yes	no	4	GCA_008042775
					Drosophila punjabiensis	yes	yes	37	GCA_008042585; SRR9997923
					Drosophila rufa	yes	no	59	GCA_018153105
					Drosophila seguyi	yes	no	151	GCA_008042675

					Drosophila serrata	yes	no	11	GCA_002093755
					Drosophila tani	yes	no	89	GCA_008042535
Drosophilinae	Drosophilini	*Drosophila*	*Sophophora*	*montium*	*Drosophila trapezifrons*	yes	no	9	GCA_018874455
					Drosophila triauraria	yes	yes	157	GCA_014170255; SRR9997941
					Drosophila truncata	yes	yes	250	GCA_008042515; SRR9997922
					Drosophila tsacasi	yes	no	8	GCA_018904565
					Drosophila vulcana	yes	no	32	GCA_008042555
					Drosophila watanabei	yes	yes	58	GCA_008042575; SRR9997920
				obscura	*Drosophila ambigua*	yes	yes	10	GCA_018150905; SRR13070667
					Drosophila athabasca	yes	yes	508	GCA_008121215; SRR9967641
					Drosophila azteca	yes	yes	281	GCA_005876895; SRR12849542
					Drosophila bifasciata	yes	no	197	GCA_009664405
					Drosophila guanche	yes	no	35	GCA_900245975
					Drosophila lowei	yes	yes	495	GCA_008121275; SRR9967667
					Drosophila miranda	yes	yes	588	GCA_003369915; SRR9211916
					Drosophila obscura	yes	no	247	GCA_018151105
					Drosophila persimilis	yes	yes	352	GCA_003286085; SRR6425998
					Drosophila pseudoobscura	yes	yes	256	GCA_009870125; SRR18151028
					Drosophila subobscura	yes	no	228	GCA_008121235
					Drosophila tristis	yes	no	213	GCA_018150885

				saltans	*Drosophila neocordata*	yes	no	158	GCA_018903615; SRR13070675
					Drosophila prosaltans	yes	yes	312	GCA_018151275; SRR13070674
					Drosophila saltans	yes	yes	392	GCA_018903575; SRR13070673
					Drosophila sturtevanti	yes	yes	801	GCA_018150375; SRR13070633
				setifemur	*Drosophila setifemur*	yes	no	14	GCA_021224005
				willistoni	*Drosophila equinoxialis*	yes	no	507	GCA_018150345
					Drosophila insularis	yes	no	383	GCA_018903935
Drosophilinae	Drosophilini	*Drosophila*	*Sophophora*	*willistoni*	*Drosophila paulistorum*	yes	no	683	GCA_018152135
					Drosophila sucinea	yes	yes	225	GCA_018150745; SRR13070638
					Drosophila tropicalis	yes	no	569	GCA_018151085
					Drosophila willistoni	yes	yes	762	GCA_018902025; SRR13703952
		Lordiphosa	--	--	*Lordiphosa mommai*	yes	no	22	GCA_018904225
				fenastrarum	*Lordiphosa collinella*	yes	yes	681	GCA_018904265; SRR13070700
				miki	*Lordiphosa clarofinis*	yes	no	14	GCA_018904275
					Lordiphosa magnipectinata	yes	no	4	GCA_018904285
					Lordiphosa stackelbergi	yes	yes	46	GCA_018904235; SRR13070699
		Scaptomyza	*Hemiscaptomyza*	--	*Scaptomyza hsui*	yes	no	75	GCA_018152825
			Parascaptomyza	--	*Scaptomyza pallida*	yes	no	151	GCA_018152965
			Scaptomyza	--	*Scaptomyza flava*	yes	no	35	GCA_003952975
				--	*Scaptomyza graminum*	yes	no	41	GCA_018901835
				--	*Scaptomyza montana*	yes	no	88	GCA_018904305

		Zaprionus	*Anaprionus*	--	*Zaprionus bogoriensis*	yes	no	35	GCA_021223985
			Zaprionus	*armatus*	*Zaprionus africanus*	yes	no	35	GCA_018151435
					Zaprionus camerounensis	yes	no	67	GCA_018904165
					Zaprionus capensis	yes	no	339	GCA_018903675
					Zaprionus davidi	yes	no	52	GCA_018903715
					Zaprionus gabonicus	yes	no	61	GCA_018903695
					Zaprionus indianus	yes	no	71	GCA_018904595
					Zaprionus lachaisei	yes	no	71	GCA_018901815
					Zaprionus nigranus	yes	no	84	GCA_018903425
					Zaprionus ornatus	yes	no	20	GCA_018904035
					Zaprionus taronus	yes	no	65	GCA_018901805
					Zaprionus tsacasi	yes	no	58	GCA_018904105
					Zaprionus vittiger	yes	no	166	GCA_018904025
Drosophilinae	Drosophilini	*Zaprionus*	*Zaprionus*	*inermis*	*Zaprionus ghesquierei*	yes	no	46	GCA_018904095
					Zaprionus inermis	yes	no	6	GCA_018151445
					Zaprionus kolodkinae	yes	no	40	GCA_018901885
Steganinae	Gitonini	*Phortica*	*Phortica*	--	*Phortica variegata*	yes	no	22	GCA_001014415
	Steganini	*Leucophenga*	--	--	*Leucophenga varia*	yes	no	44	GCA_018903435

TPase = transposase. * The genome of *Drosophila buzzatii* was downloaded from the Drosophila buzzatii Genome Project webpage (https://dbuz.uab.cat/).
