# Supplementary material for: Horizontal transfer and the widespread presence of Galileo transposons in Drosophilidae (Insecta: Diptera)
Source: Genet Mol Biol. 2024 Mar 29;46(3 Suppl 1):e20230143. doi: 10.1590/1678-4685-GMB-2023-0143 (PMC10990002; doi:10.1590/1678-4685-GMB-2023-0143)
Supplement: Table S2 - [file 1415-4757-GMB-46-3-s1-e20230143-s9.pdf]

Genetics and Molecular Biology
https://doi.org/10.1590/1678-4685-GMB-2023-0143

Supplementary Material to "Horizontal transfer and the widespread presence of *Galileo* transposons in Drosophilidae (Insecta: Diptera)"

Table S2 – List of genes used to normalize the results of profile and abundance of *Galileo* across the analyzed genomes in this study.

Gene code	Description	OrthoDB url
29at7174	Ryanodine receptor	https://www.orthodb.org/v10?query=29at7147
169at7174	Laminin, N-terminal	https://www.orthodb.org/v10?query=169at7147
516at7147	Ubiquitin carboxyl-terminal hydrolase 34	https://www.orthodb.org/v10?query=516at7147
591at7174	protein pecanex	https://www.orthodb.org/v10?query=591at7147
608at7174	EF-hand domain	https://www.orthodb.org/v10?query=608at7147
