# Supplementary material for: Horizontal transfer and the widespread presence of Galileo transposons in Drosophilidae (Insecta: Diptera)
Source: Genet Mol Biol. 2024 Mar 29;46(3 Suppl 1):e20230143. doi: 10.1590/1678-4685-GMB-2023-0143 (PMC10990002; doi:10.1590/1678-4685-GMB-2023-0143)
Supplement: Table S3 - [file 1415-4757-GMB-46-3-s1-e20230143-s10.pdf]

Genetics and Molecular Biology
https://doi.org/10.1590/1678-4685-GMB-2023-0143

Supplementary Material to "Horizontal transfer and the widespread presence of *Galileo* transposons in Drosophilidae (Insecta: Diptera)"

Table S3 – List of genes used for Codon Usage Bias (CUB) comparisons in the analysis of horizontal transposon transfer (HTT) in *vhica* R package.

Gene code	Description	OrthoDB url
10627at7147	NADH:ubiquinone oxidoreductase, subunit G	https://www.orthodb.org/v10?query=10627at7147
12214at7147	Segmentation protein cap'n'collar	https://www.orthodb.org/v10?query=12214at7147
1957at7147	Rho GTPase-activating protein domain	https://www.orthodb.org/v10?query=1957at7147
21668at7147	translation initiation factor eIF-2B subunit beta	https://www.orthodb.org/v10?query=21668at7147
23821at7147	Cytochrome P450	https://www.orthodb.org/v10?query=23821at7147
27633at7147	uncharacterized protein LOC108041683	https://www.orthodb.org/v10?query=27633at7147
28148at7147	Zona pellucida domain	https://www.orthodb.org/v10?query=28148at7147
28571at7147	tether containing UBX domain for GLUT4	https://www.orthodb.org/v10?query=28571at7147
32701at7147	Protein phosphatase methylesterase 1	https://www.orthodb.org/v10?query=32701at7147
34897at7147	WSCD family member CG9164	https://www.orthodb.org/v10?query=34897at7147
38158at7147	Ras-associating (RA) domain	https://www.orthodb.org/v10?query=38158at7147
39801at7147	Aminoacyl-tRNA synthetase, class II	https://www.orthodb.org/v10?query=39801at7147
42904at7147	Soluble NSF attachment protein	https://www.orthodb.org/v10?query=42904at7147
44372at7147	decaprenyl-diphosphate synthase subunit 1	https://www.orthodb.org/v10?query=44372at7147
47465at7147	CRAL-TRIO lipid binding domain	https://www.orthodb.org/v10?query=47465at7147
47948at7147	membrane-associated tyrosine- and threonine-specific cdc2-inhibitory kinase	https://www.orthodb.org/v10?query=47948at7147
51631at7147	OCIA domain-containing protein 1	https://www.orthodb.org/v10?query=51631at7147
53617at7147	dnaJ homolog subfamily C member 17	https://www.orthodb.org/v10?query=53617at7147
55696at7147	transmembrane protein 26	https://www.orthodb.org/v10?query=55696at7147
59202at7147	syntaxin-18	https://www.orthodb.org/v10?query=59202at7147
59792at7147	Golgi SNAP receptor complex member 1	https://www.orthodb.org/v10?query=59792at7147
60696at7147	PITH domain	https://www.orthodb.org/v10?query=60696at7147
62037at7147	vacuolar protein sorting-associated protein VTA1 homolog	https://www.orthodb.org/v10?query=62037at7147
63398at7147	class E basic helix-loop-helix protein 22	https://www.orthodb.org/v10?query=63398at7147
65583at7147	nuclear speckle splicing regulatory protein 1	https://www.orthodb.org/v10?query=65583at7147

67720at7147	Mediator of RNA polymerase II transcription subunit 10	https://www.orthodb.org/v10?query=67720at7147
68787at7147	beclin 1-associated autophagy-related key regulator	https://www.orthodb.org/v10?query=68787at7147
71857at7147	U6 snRNA-associated Sm-like protein LSm7	https://www.orthodb.org/v10?query=71857at7147
7282at7147	Armadillo	https://www.orthodb.org/v10?query=7282at7147
74859at7147	ATP synthase-coupling factor 6, mitochondrial	https://www.orthodb.org/v10?query=74859at7147
