# Supplementary material for: Horizontal transfer and the widespread presence of Galileo transposons in Drosophilidae (Insecta: Diptera)
Source: Genet Mol Biol. 2024 Mar 29;46(3 Suppl 1):e20230143. doi: 10.1590/1678-4685-GMB-2023-0143 (PMC10990002; doi:10.1590/1678-4685-GMB-2023-0143)
Supplement: Table S4 - [file 1415-4757-GMB-46-3-s1-e20230143-s11.pdf]

Genetics and Molecular Biology
https://doi.org/10.1590/1678-4685-GMB-2023-0143

Supplementary Material to "Horizontal transfer and the widespread presence of *Galileo* transposons in Drosophilidae (Insecta: Diptera)"

Table S4 – Statistics of assembly completeness for each analyzed genome.

	BUSCO statistics					
**Species**	**C**	**S**	**D**	**F**	**M**	**Assembly length (bp)**
Chymomyza costata	99.0%	98.2%	0.8%	0.2%	0.8%	315184827
Drosophila albomicans	98.9%	98.3%	0.6%	0.4%	0.7%	167541436
Drosophila ambigua	98.3%	97.7%	0.6%	0.7%	1.0%	161264264
Drosophila americana	99.0%	98.5%	0.5%	0.5%	0.5%	183572841
Drosophila ananassae	99.1%	98.8%	0.3%	0.4%	0.5%	213833366
Drosophila anomelani	95.2%	94.5%	0.7%	2.3%	2.5%	137208091
Drosophila arawakana	98.8%	97.8%	1.0%	0.4%	0.8%	171389560
Drosophila arizonae	95.3%	95.0%	0.3%	1.1%	3.6%	141386800
Drosophila asahinai	98.1%	97.2%	0.9%	0.9%	1.0%	189050820
Drosophila athabasca	98.3%	97.4%	0.9%	0.6%	1.1%	192660667
Drosophila auraria	97.7%	97.1%	0.6%	1.2%	1.1%	197420731
Drosophila azteca	98.2%	97.3%	0.9%	0.5%	1.3%	219083522
Drosophila baimaii	88.3%	86.8%	1.5%	6.2%	5.5%	175823710
Drosophila bakoue	97.9%	95.8%	2.1%	0.9%	1.2%	187248584
Drosophila barbarae	88.9%	88.4%	0.5%	6.1%	5.0%	113181336
Drosophila biarmipes	98.9%	98.7%	0.2%	0.5%	0.6%	185318521
Drosophila bifasciata	92.6%	92.1%	0.5%	1.8%	5.6%	192749618
Drosophila bipectinata	99.1%	98.5%	0.6%	0.4%	0.5%	192393922
Drosophila birchii	98.6%	98.0%	0.6%	0.5%	0.9%	156593892
Drosophila bocki	98.1%	97.3%	0.8%	0.9%	1.0%	151202254
Drosophila bocqueti	98.3%	97.4%	0.9%	0.8%	0.9%	173041441
Drosophila bunnanda	98.7%	98.1%	0.6%	0.6%	0.7%	156309106
Drosophila burlai	97.7%	94.8%	2.9%	1.2%	1.1%	175666184
Drosophila busckii	97.4%	96.7%	0.7%	0.6%	2.0%	118492362
Drosophila buzzatii	98.2%	98.0%	0.2%	0.9%	0.9%	161490851

Drosophila cardini	98.4%	97.7%	0.7%	0.7%	0.9%	181105816
Drosophila carrolli	98.3%	97.9%	0.4%	0.6%	1.1%	231219246
Drosophila chauvacae	91.3%	90.8%	0.5%	4.7%	4.0%	133641973
Drosophila diplacantha	86.6%	85.8%	0.8%	8.0%	5.4%	117734998
Drosophila dunni	98.9%	98.5%	0.4%	0.3%	0.8%	177495819
Drosophila elegans	99.1%	98.9%	0.2%	0.5%	0.4%	178445117
Drosophila equinoxialis	97.3%	96.1%	1.2%	1.4%	1.3%	221733993
Drosophila ercepeae	99.1%	98.8%	0.3%	0.4%	0.5%	178957960
Drosophila erecta	99.2%	98.8%	0.4%	0.3%	0.5%	146538397
Drosophila eugracilis	98.5%	98.0%	0.5%	0.8%	0.7%	164811307
Drosophila fengkainensis	79.3%	78.4%	0.9%	11.6%	9.1%	158250599
Drosophila ficusphila	98.7%	98.5%	0.2%	0.8%	0.5%	167832931
Drosophila funebris	98.2%	98.0%	0.2%	0.8%	1.0%	154245976
Drosophila fuyamai	99.1%	98.5%	0.6%	0.5%	0.4%	229100662
Drosophila greeni	71.4%	70.9%	0.5%	10.0%	18.6%	112789423
Drosophila grimshawi	99.1%	98.5%	0.6%	0.3%	0.6%	191382978
Drosophila guanche	98.9%	98.4%	0.5%	0.6%	0.5%	140653667
Drosophila gunungcola	98.7%	98.5%	0.2%	0.5%	0.8%	168095110
Drosophila hydei	98.9%	97.0%	1.9%	0.5%	0.6%	153741574
Drosophila immigrans	99.0%	98.4%	0.6%	0.5%	0.5%	183277074
Drosophila innubila	98.9%	98.4%	0.5%	0.4%	0.7%	166284835
Drosophila insularis	99.2%	98.5%	0.7%	0.4%	0.4%	212411654
Drosophila ironensis	98.6%	97.4%	1.2%	0.6%	0.8%	144278514
Drosophila jambulina	98.9%	98.3%	0.6%	0.5%	0.6%	179653633
Drosophila kanapiae	98.8%	97.9%	0.9%	0.6%	0.6%	152203088
Drosophila kikkawai	98.8%	97.9%	0.9%	0.5%	0.7%	188799863
Drosophila kohkoa	98.8%	92.4%	6.4%	0.6%	0.6%	170668061
Drosophila kurseongensis	98.9%	98.6%	0.3%	0.5%	0.6%	206440819
Drosophila lacertosa	98.5%	98.2%	0.3%	0.8%	0.7%	154130906
Drosophila lacteicornis	98.1%	97.0%	1.1%	1.4%	0.5%	182681050
Drosophila leontia	97.3%	94.3%	3.0%	1.6%	1.1%	164601511
Drosophila lini	94.9%	94.2%	0.7%	3.1%	2.0%	133806944
Drosophila littoralis	99.0%	98.7%	0.3%	0.5%	0.5%	236786485
Drosophila lowei	95.6%	94.4%	1.2%	1.2%	3.2%	184313494

Drosophila malagassya	93.1%	92.4%	0.7%	3.8%	3.1%	143663510
Drosophila malerkotliana	99.0%	98.5%	0.5%	0.5%	0.5%	191313204
Drosophila mauritiana	99.0%	98.8%	0.2%	0.5%	0.5%	152317181
Drosophila mayri	98.8%	98.0%	0.8%	0.4%	0.8%	167807061
Drosophila melanica	97.0%	96.7%	0.3%	1.6%	1.4%	149951920
Drosophila melanogaster	98.6%	98.4%	0.2%	0.5%	0.9%	143726002
Drosophila micromelanica	97.7%	97.5%	0.2%	1.2%	1.1%	156838486
Drosophila miranda	98.9%	82.6%	16.3%	0.8%	0.3%	287096000
Drosophila mojavensis	99.0%	98.6%	0.4%	0.3%	0.7%	163170721
Drosophila montana	95.2%	94.8%	0.4%	2.6%	2.2%	183585048
Drosophila murphyi	99.1%	98.6%	0.5%	0.3%	0.6%	153852709
Drosophila nannoptera	98.1%	97.3%	0.8%	0.7%	1.2%	134504500
Drosophila nasuta	99.2%	98.4%	0.8%	0.2%	0.6%	173158203
Drosophila navojoa	98.1%	97.8%	0.3%	0.9%	1.0%	147357603
Drosophila neocordata	99.3%	98.4%	0.9%	0.2%	0.5%	194830689
Drosophila neonasuta	78.4%	77.8%	0.6%	9.6%	12.0%	131273702
Drosophila nigrodunni	98.2%	97.9%	0.3%	0.5%	1.3%	145305457
Drosophila nigromelanica	98.2%	97.9%	0.3%	1.0%	0.8%	162543777
Drosophila nikananu	98.0%	96.0%	2.0%	1.1%	0.9%	190505469
Drosophila novamexicana	98.2%	97.4%	0.8%	0.4%	1.4%	177223868
Drosophila obscura	98.4%	97.6%	0.8%	0.9%	0.7%	179834232
Drosophila ogumai	96.2%	95.6%	0.6%	2.2%	1.6%	134345277
Drosophila ohnishii	90.6%	90.0%	0.6%	5.5%	3.9%	133626863
Drosophila orena	97.4%	93.2%	4.2%	0.5%	2.1%	182891478
Drosophila orosa	94.7%	94.0%	0.7%	3.2%	2.1%	142361551
Drosophila oshimai	98.4%	97.6%	0.8%	0.9%	0.7%	181013079
Drosophila pachea	98.7%	98.4%	0.3%	0.5%	0.8%	127516150
Drosophila pandora	99.0%	95.1%	3.9%	0.4%	0.6%	181393585
Drosophila parabipectinata	99.1%	98.6%	0.5%	0.4%	0.5%	209862113
Drosophila parvula	90.9%	90.4%	0.5%	5.1%	4.0%	143573646
Drosophila paulistorum	99.1%	98.2%	0.9%	0.3%	0.6%	321171226
Drosophila pectinifera	98.5%	98.0%	0.5%	0.7%	0.8%	149209000
Drosophila persimilis	98.9%	97.2%	1.7%	0.8%	0.3%	195512972
Drosophila prosaltans	99.0%	98.2%	0.8%	0.3%	0.7%	214135114

Drosophila pruinosa	98.9%	98.4%	0.5%	0.5%	0.6%	201065926
Drosophila pseudoananassae	98.5%	98.0%	0.5%	0.8%	0.7%	193740970
Drosophila pseudoobscura	98.7%	98.0%	0.7%	0.9%	0.4%	163282969
Drosophila pseudotakahashii	99.1%	98.6%	0.5%	0.4%	0.5%	185796206
Drosophila punjabiensis	98.3%	93.6%	4.7%	0.9%	0.8%	192339030
Drosophila quadrilineata	98.9%	98.5%	0.4%	0.3%	0.8%	202639777
Drosophila repleta	98.9%	98.7%	0.2%	0.5%	0.6%	154011936
Drosophila repletoides	99.3%	98.4%	0.9%	0.2%	0.5%	181448104
Drosophila rhopaloa	98.5%	98.1%	0.4%	0.7%	0.8%	193508231
Drosophila robusta	94.9%	94.4%	0.5%	2.6%	2.5%	179073841
Drosophila rubida	98.2%	97.8%	0.4%	0.3%	1.5%	137777123
Drosophila rufa	98.7%	97.8%	0.9%	0.5%	0.8%	208439571
Drosophila saltans	99.2%	98.4%	0.8%	0.4%	0.4%	249306946
Drosophila santomea	99.1%	98.9%	0.2%	0.3%	0.6%	146816533
Drosophila sechellia	99.1%	98.7%	0.4%	0.4%	0.5%	153103154
Drosophila seguyi	97.9%	96.8%	1.1%	0.8%	1.3%	178856532
Drosophila serrata	97.3%	95.4%	1.9%	1.7%	1.0%	198035861
Drosophila setifemur	99.0%	98.5%	0.5%	0.4%	0.6%	155894680
Drosophila simulans	99.0%	98.8%	0.2%	0.4%	0.6%	131682525
Drosophila sproati	99.1%	98.6%	0.5%	0.2%	0.7%	138134348
Drosophila sturtevanti	98.8%	97.9%	0.9%	0.5%	0.7%	172592868
Drosophila subobscura	98.7%	98.2%	0.5%	0.7%	0.6%	126232139
Drosophila subpulchrella	98.9%	93.8%	5.1%	0.6%	0.5%	270691342
Drosophila sucinea	98.3%	97.8%	0.5%	0.6%	1.1%	184689537
Drosophila sulfurigaster	97.2%	96.6%	0.6%	0.5%	2.3%	158690976
Drosophila suzukii	97.4%	94.6%	2.8%	1.5%	1.1%	268012156
Drosophila takahashii	97.8%	97.4%	0.4%	0.5%	1.7%	165528346
Drosophila tani	97.7%	94.7%	3.0%	1.1%	1.2%	180972673
Drosophila teissieri	99.0%	98.1%	0.9%	0.5%	0.5%	149510488
Drosophila trapezifrons	83.8%	83.3%	0.5%	9.2%	7.0%	151600198
Drosophila triauraria	92.2%	91.3%	0.9%	1.8%	6.0%	195275685
Drosophila tristis	97.2%	96.7%	0.5%	0.9%	1.9%	158167647
Drosophila tropicalis	98.2%	97.2%	1.0%	0.9%	0.9%	211041385
Drosophila truncata	98.5%	97.9%	0.6%	0.7%	0.8%	167897087

Drosophila tsacasi	92.7%	92.1%	0.6%	3.9%	3.4%	144985661
Drosophila varians	98.7%	98.5%	0.2%	0.7%	0.6%	143042420
Drosophila virilis	99.0%	97.2%	1.8%	0.5%	0.5%	189443829
Drosophila vulcana	98.3%	97.4%	0.9%	1.0%	0.7%	187578810
Drosophila watanabei	97.9%	91.6%	6.3%	1.3%	0.8%	196825890
Drosophila willistoni	98.8%	97.7%	1.1%	0.3%	0.9%	246985538
Drosophila yakuba	99.0%	98.8%	0.2%	0.4%	0.6%	147899121
Leucophenga varia	94.5%	90.1%	4.4%	2.2%	3.3%	303593356
Lordiphosa clarofinis	98.0%	94.9%	3.1%	0.7%	1.3%	398719931
Lordiphosa collinella	96.3%	93.6%	2.7%	0.9%	2.8%	375096912
Lordiphosa magnipectinata	97.0%	94.2%	2.8%	1.2%	1.8%	416514997
Lordiphosa mommai	97.0%	95.3%	1.7%	0.7%	2.3%	339463949
Lordiphosa stackelbergi	77.0%	75.1%	1.9%	2.6%	20.4%	307819360
Phortica variegata	94.0%	93.6%	0.4%	2.9%	3.1%	155550413
Scaptodrosophila lebanonensis	98.9%	97.7%	1.2%	0.5%	0.6%	247077949
Scaptomyza flava	98.1%	94.0%	4.1%	0.9%	1.0%	214836881
Scaptomyza graminum	98.9%	98.2%	0.7%	0.3%	0.8%	137787325
Scaptomyza hsui	98.5%	97.7%	0.8%	0.5%	1.0%	223546733
Scaptomyza montana	98.7%	97.8%	0.9%	0.6%	0.7%	229076798
Scaptomyza pallida	97.7%	96.4%	1.3%	1.2%	1.1%	201650218
Zaprionus africanus	99.0%	98.4%	0.6%	0.3%	0.7%	162493599
Zaprionus bogoriensis	99.0%	98.7%	0.3%	0.5%	0.5%	158470898
Zaprionus camerounensis	98.9%	98.2%	0.7%	0.3%	0.8%	167162588
Zaprionus capensis	99.0%	97.8%	1.2%	0.4%	0.6%	166762837
Zaprionus davidi	99.1%	98.0%	1.1%	0.3%	0.6%	156049829
Zaprionus gabonicus	99.0%	98.5%	0.5%	0.3%	0.7%	187602806
Zaprionus ghesquierei	99.2%	97.9%	1.3%	0.2%	0.6%	198077797
Zaprionus indianus	98.6%	98.1%	0.5%	0.5%	0.9%	197260855
Zaprionus inermis	99.0%	98.5%	0.5%	0.4%	0.6%	149549994
Zaprionus kolodkinae	98.8%	98.3%	0.5%	0.6%	0.6%	182973347
Zaprionus lachaisei	98.2%	97.7%	0.5%	0.9%	0.9%	148387969
Zaprionus nigranus	96.4%	94.8%	1.6%	1.8%	1.8%	175473336
Zaprionus ornatus	98.9%	97.7%	1.2%	0.5%	0.6%	206772757

Zaprionus taronus	96.8%	95.6%	1.2%	1.8%	1.4%	162912716
Zaprionus tsacasi	98.7%	98.3%	0.4%	0.5%	0.8%	165518990
Zaprionus vittiger	99.0%	98.5%	0.5%	0.4%	0.6%	162326431

C = complete; S = complete and single copy; D = duplicated; F = fragmented; M = missing.
