# Supplementary material for: Horizontal transfer and the widespread presence of Galileo transposons in Drosophilidae (Insecta: Diptera)
Source: Genet Mol Biol. 2024 Mar 29;46(3 Suppl 1):e20230143. doi: 10.1590/1678-4685-GMB-2023-0143 (PMC10990002; doi:10.1590/1678-4685-GMB-2023-0143)
Supplement: Table S5 - [file 1415-4757-GMB-46-3-s1-e20230143-s12.pdf]

Genetics and Molecular Biology
https://doi.org/10.1590/1678-4685-GMB-2023-0143

Supplementary Material to "Horizontal transfer and the widespread presence of *Galileo* transposons in Drosophilidae (Insecta: Diptera)"

Table S5 – Statistically significant results of pairwise comparisons of horizontal transposon transfer performed with *vhica* at HTT-DB.

seq	dS	sp1	sp2	Time(Mya)	p-value
Galileo.a.b	0.036523000	daur.a	lcol.b	1.141344000	0.040791490
Galileo.a	0.052293610	daur.a	lsta.a	1.634175000	0.010181530
Galileo.a.c	0.125107900	daur.a	drho.c	3.909621000	0.021880570
Galileo.a.b	0.127005000	daur.a	drho.b	3.968906000	0.026963370
Galileo.a	0.128377600	daur.a	drho.a	4.011799000	0.038896610
Galileo.a.b	0.133094800	daur.a	dcar.b	4.159212000	0.047961980
Galileo.a.d	0.143744000	daur.a	drho.d	4.492000000	0.043489600
Galileo.b.a	0.021728160	daur.b	lsta.a	0.679005100	0.005497651
Galileo.b	0.043909280	daur.b	lcol.b	1.372165000	0.040179700
Galileo.b	0.127276100	daur.b	drho.b	3.977377000	0.024015080
Galileo.b.c	0.135754600	daur.b	drho.c	4.242331000	0.027018160
Galileo.b.a	0.139280800	daur.b	drho.a	4.352524000	0.048117230
Galileo.a.b	0.098775170	dcar.a	dtri.b	3.086724000	0.018260640
Galileo.a	0.125295600	dcar.a	dpun.a	3.915486000	0.044974350
Galileo.a	0.132991700	dcar.a	djam.a	4.155989000	0.005236886
Galileo.a	0.136929700	dcar.a	dleo.a	4.279055000	0.025842740
Galileo.a.d	0.137906200	dcar.a	djam.d	4.309568000	0.008303978
Galileo.a.b	0.140858000	dcar.a	djam.b	4.401813000	0.012641330
Galileo.a.c	0.147227600	dcar.a	djam.c	4.600862000	0.015060720
Galileo.a	0.148268400	dcar.a	dmay.a	4.633388000	0.024091360
Galileo.a	0.153466700	dcar.a	dwat.a	4.795834000	0.011107040
Galileo.a	0.154377900	dcar.a	dtru.a	4.824310000	0.001706711
Galileo.a.b	0.184329300	dcar.a	dwat.b	5.760290000	0.032432140
Galileo.b	0.101492200	dcar.b	dtri.b	3.171631000	0.010333520
Galileo.b.a	0.123791300	dcar.b	dpun.a	3.868477000	0.025375700
Galileo.b.a	0.135014300	dcar.b	djam.a	4.219197000	0.002746353
Galileo.b.a	0.139232300	dcar.b	dleo.a	4.351008000	0.016572120
Galileo.b.d	0.139848200	dcar.b	djam.d	4.370257000	0.004398396
Galileo.b	0.142992100	dcar.b	djam.b	4.468504000	0.006818641
Galileo.b.c	0.149163200	dcar.b	djam.c	4.661349000	0.008110761
Galileo.b.a	0.151703400	dcar.b	dwat.a	4.740731000	0.006345174
Galileo.b.a	0.154439500	dcar.b	dmay.a	4.826235000	0.017349430
Galileo.b.a	0.156947900	dcar.b	dtru.a	4.904623000	0.000905884
Galileo.b	0.178332700	dcar.b	dwat.b	5.572897000	0.016624520
Galileo.c.b	0.113482600	dcar.c	dtri.b	3.546331000	0.026070960
Galileo.c.a	0.143241500	dcar.c	djam.a	4.476296000	0.006500824
Galileo.c.d	0.148328500	dcar.c	djam.d	4.635267000	0.010321500
Galileo.c.b	0.150886800	dcar.c	djam.b	4.715214000	0.015452800
Galileo.c.a	0.154405800	dcar.c	dleo.a	4.825182000	0.039901330
Galileo.c	0.157938000	dcar.c	djam.c	4.935561000	0.018780970
Galileo.c.a	0.170630700	dcar.c	dmay.a	5.332210000	0.041424030

seq	dS	sp1	sp2	Time(Mya)	p-value
Galileo.c.a	0.171645100	dcar.c	dwat.a	5.363909000	0.018360470
Galileo.c.a	0.181305200	dcar.c	dtru.a	5.665787000	0.003888839
Galileo.c.b	0.194250700	dcar.c	dwat.b	6.070334000	0.040579220
Galileo.d.b	0.094335750	dcar.d	dtri.b	2.947992000	0.011664370
Galileo.d.a	0.119140300	dcar.d	dpun.a	3.723134000	0.029675860
Galileo.d.a	0.120032600	dcar.d	djam.a	3.751018000	0.002420138
Galileo.d	0.124619800	dcar.d	djam.d	3.894369000	0.003853000
Galileo.d.c	0.134018200	dcar.d	djam.c	4.188069000	0.007149270
Galileo.d.b	0.144634800	dcar.d	djam.b	4.519839000	0.010433370
Galileo.d.a	0.147242000	dcar.d	dwat.a	4.601313000	0.007172570
Galileo.d.a	0.147596200	dcar.d	dmay.a	4.612381000	0.018713550
Galileo.d.a	0.157142700	dcar.d	dtru.a	4.910708000	0.001351487
Galileo.d.b	0.178196500	dcar.d	dwat.b	5.568641000	0.021465120
Galileo.e.b	0.099488540	dcar.e	dtri.b	3.109017000	0.021031420
Galileo.e.a	0.133834800	dcar.e	djam.a	4.182336000	0.006123647
Galileo.e.a	0.134074600	dcar.e	dleo.a	4.189830000	0.026008740
Galileo.e.d	0.138805100	dcar.e	djam.d	4.337659000	0.009697468
Galileo.e.b	0.141738800	dcar.e	djam.b	4.429338000	0.014710910
Galileo.e.c	0.148154400	dcar.e	djam.c	4.629825000	0.017530390
Galileo.e.a	0.149173600	dcar.e	dmay.a	4.661674000	0.027088860
Galileo.e.a	0.154519600	dcar.e	dwat.a	4.828738000	0.012581600
Galileo.e.a	0.155391700	dcar.e	dtru.a	4.855990000	0.002016841
Galileo.e.b	0.185533600	dcar.e	dwat.b	5.797924000	0.036680790
Galileo.a.b	0.090551920	dfuy.a	dtri.b	2.829748000	0.017009320
Galileo.a	0.124130300	dfuy.a	djam.a	3.879073000	0.008950692
Galileo.a	0.128143300	dfuy.a	dleo.a	4.004479000	0.026569030
Galileo.a.d	0.128777400	dfuy.a	djam.d	4.024292000	0.013300690
Galileo.a.b	0.131953400	dfuy.a	djam.b	4.123545000	0.019248420
Galileo.a.c	0.138089800	dfuy.a	djam.c	4.315308000	0.022718460
Galileo.a	0.144132600	dfuy.a	dwat.a	4.504144000	0.044059600
Galileo.a	0.152790400	dfuy.a	dtru.a	4.774699000	0.009344020
Galileo.b	0.093906950	dfuy.b	dtri.b	2.934592000	0.019141790
Galileo.b.a	0.127113100	dfuy.b	djam.a	3.972286000	0.009945961
Galileo.b.a	0.131059100	dfuy.b	dleo.a	4.095597000	0.029262040
Galileo.b.d	0.131817700	dfuy.b	djam.d	4.119304000	0.014778560
Galileo.b	0.134983000	dfuy.b	djam.b	4.218220000	0.021341090
Galileo.b.c	0.141102000	dfuy.b	djam.c	4.409436000	0.025154150
Galileo.b.a	0.147125300	dfuy.b	dwat.a	4.597664000	0.048186400
Galileo.b.a	0.156273200	dfuy.b	dtru.a	4.883537000	0.010520910
Galileo.c.b	0.105205200	dfuy.c	dtri.b	3.287664000	0.028578520
Galileo.c.a	0.138983500	dfuy.c	djam.a	4.343233000	0.015083290
Galileo.c.a	0.143007500	dfuy.c	dleo.a	4.468983000	0.043276160
Galileo.c.d	0.143917800	dfuy.c	djam.d	4.497432000	0.022401410
Galileo.c.b	0.146727300	dfuy.c	djam.b	4.585229000	0.031769690
Galileo.c	0.153149600	dfuy.c	djam.c	4.785925000	0.037668760
Galileo.c.a	0.168641600	dfuy.c	dtru.a	5.270050000	0.016031890
Galileo.d.b	0.103675500	dfuy.d	dtri.b	3.239860000	0.026215410
Galileo.d.a	0.136779200	dfuy.d	djam.a	4.274349000	0.013572950
Galileo.d.a	0.140790000	dfuy.d	dleo.a	4.399687000	0.039402360
Galileo.d	0.141530200	dfuy.d	djam.d	4.422819000	0.020083360
Galileo.d.b	0.144935100	dfuy.d	djam.b	4.529222000	0.029058430
Galileo.d.c	0.150871900	dfuy.d	djam.c	4.714748000	0.033977860
Galileo.d.a	0.166144100	dfuy.d	dtru.a	5.192002000	0.014374130

seq	dS	sp1	sp2	Time(Mya)	p-value
Galileo.e.a	0.173538300	dfuy.e	djam.a	5.423072000	0.045362350
Galileo.e.a	0.188572300	dfuy.e	dtru.a	5.892885000	0.030562590
Galileo.f.a	0.128188900	dfuy.f	djam.a	4.005903000	0.010650910
Galileo.f.a	0.132102500	dfuy.f	dleo.a	4.128204000	0.030995990
Galileo.f.d	0.132965900	dfuy.f	djam.d	4.155184000	0.015842470
Galileo.f.b	0.135909200	dfuy.f	djam.b	4.247162000	0.022693840
Galileo.f.c	0.142239700	dfuy.f	djam.c	4.444992000	0.026909240
Galileo.f.a	0.157478900	dfuy.f	dtru.a	4.921215000	0.011260380
Galileo.a.b	0.046858100	djam.a	lcol.b	1.464316000	0.039568460
Galileo.a.c	0.127113100	djam.a	drho.c	3.972286000	0.003539613
Galileo.a.b	0.128863200	djam.a	drho.b	4.026975000	0.004411770
Galileo.a	0.130229800	djam.a	drho.a	4.069681000	0.006545616
Galileo.a	0.135551200	djam.a	dkur.a	4.235974000	0.022141960
Galileo.a.d	0.141086900	djam.a	drho.d	4.408965000	0.006394480
Galileo.b	0.126594600	djam.b	drho.b	3.956081000	0.007712941
Galileo.b.c	0.134983000	djam.b	drho.c	4.218220000	0.008732943
Galileo.b.a	0.147420900	djam.b	drho.a	4.606903000	0.021271170
Galileo.b.d	0.166649100	djam.b	drho.d	5.207784000	0.027127320
Galileo.c.a	0.136821700	djam.c	drho.a	4.275678000	0.014694650
Galileo.c	0.141102000	djam.c	drho.c	4.409436000	0.010376730
Galileo.c.b	0.143255600	djam.c	drho.b	4.476736000	0.012973730
Galileo.c.d	0.155528600	djam.c	drho.d	4.860268000	0.018509630
Galileo.d.a	0.127398500	djam.d	drho.a	3.981202000	0.008023126
Galileo.d.c	0.131817700	djam.d	drho.c	4.119304000	0.005637697
Galileo.d.b	0.133667600	djam.d	drho.b	4.177113000	0.007020119
Galileo.d.a	0.140205800	djam.d	dkur.a	4.381430000	0.033192700
Galileo.d	0.145897800	djam.d	drho.d	4.559308000	0.010102330
Galileo.a.b	0.113184300	dkur.a	dtri.b	3.537011000	0.046394900
Galileo.a	0.132677800	dkur.a	dleo.a	4.146181000	0.041508320
Galileo.a	0.153235800	dkur.a	dmay.a	4.788619000	0.048152320
Galileo.a	0.167767000	dkur.a	dtru.a	5.242718000	0.019085460
Galileo.a	0.055896800	dleo.a	lsta.a	1.746775000	0.038363910
Galileo.a.c	0.131059100	dleo.a	drho.c	4.095597000	0.016657110
Galileo.a.b	0.132857000	dleo.a	drho.b	4.151782000	0.019810970
Galileo.a	0.133750100	dleo.a	drho.a	4.179692000	0.026254190
Galileo.a.d	0.144747100	dleo.a	drho.d	4.523348000	0.027852950
Galileo.a	0.070692560	dpun.a	lsta.a	2.209142000	0.012057580
Galileo.a.c	0.119257100	dpun.a	drho.c	3.726783000	0.020185970
Galileo.a.b	0.120563400	dpun.a	drho.b	3.767607000	0.023959620
Galileo.a	0.121938400	dpun.a	drho.a	3.810575000	0.033279480
Galileo.a.d	0.133241400	dpun.a	drho.d	4.163795000	0.033596870
Galileo.a.b	0.097043930	drho.a	dtri.b	3.032623000	0.009728666
Galileo.a	0.139278400	drho.a	dtru.a	4.352449000	0.003674504
Galileo.a	0.150132000	drho.a	dwat.a	4.691625000	0.014136030
Galileo.a	0.165984800	drho.a	dtri.a	5.187025000	0.048012570
Galileo.a.b	0.180869700	drho.a	dwat.b	5.652179000	0.040820620
Galileo.b	0.094973000	drho.b	dtri.b	2.967906000	0.006333461
Galileo.b.a	0.138866200	drho.b	dwat.a	4.339569000	0.007567211
Galileo.b.a	0.157499300	drho.b	dtri.a	4.921852000	0.025985420
Galileo.b.a	0.158843200	drho.b	dtru.a	4.963849000	0.005092722
Galileo.b	0.170153700	drho.b	dwat.b	5.317302000	0.022941940
Galileo.c.b	0.093906950	drho.c	dtri.b	2.934592000	0.005174353
Galileo.c.a	0.147125300	drho.c	dwat.a	4.597664000	0.008877549

seq	dS	sp1	sp2	Time(Mya)	p-value
Galileo.c.a	0.156273200	drho.c	dtru.a	4.883537000	0.004028957
Galileo.c.a	0.165935900	drho.c	dtri.a	5.185496000	0.029691040
Galileo.c.b	0.177623900	drho.c	dwat.b	5.550746000	0.026042150
Galileo.d.b	0.111752900	drho.d	dtri.b	3.492278000	0.011039650
Galileo.d.a	0.162130700	drho.d	dwat.a	5.066583000	0.015778340
Galileo.d.a	0.188864700	drho.d	dtru.a	5.902022000	0.012994090
Galileo.d.b	0.192989100	drho.d	dwat.b	6.030911000	0.045478570
Galileo.a	0.046927040	dtri.a	lsta.a	1.466470000	0.015778730
Galileo.b.a	0.024800410	dtri.b	lsta.a	0.775012700	0.021436890
